# The relationship between health insurance and economic performance: an empirical study based on meta-analysis

**DOI:** 10.3389/fpubh.2024.1365877

**Published:** 2024-04-03

**Authors:** Chenchen Fan, Chunyan Li, Xiaoting Song

**Affiliations:** Shanghai International College of Intellectual Property, Tongji University, Shanghai, China

**Keywords:** health insurance, economic performance, meta-analysis, the magnitude of a correlation, moderating effects

## Abstract

Health insurance stands as a pivotal facet of social wellbeing, with profound implications for the overarching landscape of economic development. The existing research, however, lacks consensus on the relationship between health insurance and economic performance and provides no evidence about the magnitude of the correlation. This lack of information seriously impedes the high-quality development of the healthcare system. Therefore, to scientifically elucidate the relationship between the two, this study involved a meta-analysis, analyzing 479 effect values derived from 34 independent research samples. The results reveal a strongly positive correlation between health insurance and economic performance [*r* = 0.429, 95% CI = (0.381, 0.475)]. Findings show that health insurance in developed countries more effectively fosters economic performance than in developing countries. Moreover, public health insurance exerts a stronger promoting effect on economic performance than commercial health insurance. The relationship between health insurance and economic performance is moderated by data type, research method, country of sample origin, literature type, journal impact factor, publication year, type of health insurance, and the research populations. Based on meta-analysis, this study not only scientifically responds to the controversy of the relationship between health insurance and economic performance, and the magnitude of a correlation, but also further reveals the inner conduction mechanism between the two. Our research findings are meaningful for policymakers to choose an appropriate healthcare strategy according to their unique attributes, propelling sustainable economic development.

## 1 Introduction

As a primary component of the health strategy, health insurance not only ensures accessibility to medical services but also contributes to improving life expectancy, and social welfare (1). It serves as a long-term safeguard mechanism, dispersing health risks and preventing a return to poverty due to illness, playing a crucial role in economic development. Implementing and enhancing health insurance strengthens the economic foundation and improves economic performance (2). For instance, the government stimulates domestic demand and enhances residents' consumption by improving medical coverage and increasing healthcare spending, replacing precautionary savings (3–5). Prior research has also shown that health insurance enhances productivity and makes human capital more valuable, leading to a positive contribution to economic growth (2, 6, 7).

With the swift evolution of the healthcare system, the relationship between health insurance and economic performance has become a hot research topic in the field of public health. In particular, it significantly impacts the sustained progress of a nation or region's healthcare system, the wellbeing of its inhabitants, and the overall economy. Therefore, exploring the relationship between the two is of significant practical importance for establishing and improving focused healthcare systems. This study focuses on the following questions: First, does health insurance promote economic performance? Second, what is the magnitude of the correlation between health insurance and economic performance? Third, why do existing empirical research conclusions differ?

While scholars have explored the impact of health insurance on economic performance from various perspectives, there is no consensus on the relationship between the two. In varied research contexts and datasets, studies have shown that the relationship between the two may be positive, negative, or uncorrelated (2, 8–10). A consensus is emerging, however, that investing in health insurance is a strategy for cultivating human capital. Some scholars contend that health insurance fosters human capital by improving educational outcomes through the development of cognitive abilities (11). Furthermore, it directly stimulates economic performance by increasing individual income through higher labor productivity, marginal productivity, working hours, and the duration of production activities (12–14). Researchers have found that health insurance significantly reduces medical expenditure risks, lowering poverty and boosting income, preventing health-related poverty for low-income groups, and enhancing residents' consumption capacity (8, 15–18). However, not all studies support the view that healthcare positively influences economic performance. Some scholars have argued that excessive healthcare spending may adversely affect the economy by crowding out tangible capital investment (12). Healthcare consumption is also driven not only by necessity but also by the desire to maintain health. This may result in excessive investment in human capital, as the demand for quality services during economic growth remains unmet, and the role of health insurance in reducing residents' catastrophic medical expenses is not fully realized (9, 10, 12, 19–22) This could imply a heightened crowding-out effect on physical capital investment. Some scholars also believe that healthcare may hinder economic growth, given that population aging often correlates with increased healthcare spending (20).

In summary, numerous empirical studies have analyzed the relationship between health insurance and economic performance, yielding valuable and reference-worthy research conclusions. However, in terms of research content and perspective, existing studies still exhibit the following shortcomings. Firstly, while the majority of literature results suggest a positive impact of healthcare insurance on economic performance, scholars in diverse research contexts have also highlighted potential negative or unrelated relationships between the two. This contradictory relationship impedes the direct application of current research findings to the development of healthcare systems. Thus, further exploration is warranted to better understand the impact of healthcare insurance on economic performance. Secondly, previous studies only focus on the impact of health insurance on economic performance, neglecting the magnitude of the correlation between the two. Given increasing national demands for healthcare quality and wellbeing, exploring the extent of the impact is crucial for formulating and implementing health service policy formulation and implementation. Thirdly, although scholars have conducted qualitative reviews and quantitative summaries of health insurance's economic performance, current research still has limitations. Subjective influences and representative biases often exist in the literature selection of qualitative studies, affecting the accuracy and objectivity of research results. In quantitative research, there were significant differences in the measurement and definition of some key variables, leading to a lack of scientific, comprehensive, and systematic studies. So, there is a pressing need for more precise and objective research methods to overcome current research bottlenecks.

To fill the above research gaps, this study aims to contribute several novel insights into the relationship between health insurance and economic performance by meta-analysis methodology. Compared with the existing literature, this study's marginal innovation lies in three aspects. First, this study provides a scientific response to contradictions in findings about the relationship between health insurance and economic performance. Through meta-analysis, we systematically summarized and further investigated existing empirical studies on the impact of health insurance on economic performance, accurately presenting the relationship between the two. This not only enhances the content and methodology of healthcare research but also provides robust empirical evidence to support high-quality development. Second, grounded in a scientific response to the relationship between health insurance and economic performance, this study aims to explore in depth the strength of the correlation. By integrating varied research samples and model factors. With a more objective and precise, understanding of the magnitude of the correlation, we provide valuable references for formulating rational health insurance strategies. Third, no attention has yet been paid to the causes of heterogeneity in the relationship between health insurance and economic performance. This study delves into the reasons for divergent research conclusions and identifies moderating variables leading to diverse research outcomes across four levels: samples, literature, measurement, and variables. Moreover, the study extensively examines scenarios in which medical insurance exhibits diverse effects on economic performance, offering references for formulating strategies and improving economic outcomes.

The research roadmap is as follows: firstly, a review of the existing relevant literature is conducted, developing hypotheses and an analytical framework. Secondly, the literature is rigorously screened and information is extracted, strictly adhering to the steps of meta-analysis, followed by effect size calculation and an exploration of heterogeneity. Thirdly, the results of the meta-analysis are conducted, including a publication bias test, overall effect and moderating effect analysis, and a robustness test. Finally, the results of this research are presented and discussed.

## 2 Research hypothesis and framework

### 2.1 The impact of health insurance on economic performance and its sub-dimensions

The distinct economic performance generated by health insurance has garnered significant attention from scholars. Scholars have primarily examined the mechanisms that underlying how health insurance impacts economic performance, from both a societal and individual perspective. At the societal level, the widespread accessibility of health insurance has profound implications for economic performance. Firstly, health insurance plays a pivotal role in shaping economic performance by mitigating inequality and enhancing labor productivity. According to Roemer's theory of equal opportunity, health disparities arising from environmental factors are deemed unjustifiable (23). Universal health insurance enables residents to share medical resources more equitably, alleviating health inequalities arising from different environmental conditions. With health insurance, people can access medical services more promptly, reducing productivity losses due to illness, contributing to the overall productivity of society, and promoting economic development (15). Health insurance also facilitates the accumulation of human and material capital within families, effectively driving investments in health and promoting the overall improvement of workforce quality (24). Secondly, health insurance can maintain economic stability. By mitigating the financial impact of medical expenses on household budgets, it curtails the financial risks of illness. This, in turn, helps in sustaining the financial stability of households and mitigating economic fluctuations triggered by medical costs. Furthermore, health insurance contributes to improving the overall health status of society, curbing the spread of diseases. When health levels are higher, disruptions in the labor force are minimized and stability in the labor market is fostered. These combined factors actively propel sustainable economic development, establishing a more stable and dependable economic environment (25). Thirdly, health insurance alleviates fiscal pressure. By making early treatment and chronic disease management affordable for people, health insurance alleviates long-term medical expenses, providing a buffer for public finances (26). Fourthly, health insurance contributes to maintaining social order, which in turn affects economic performance. It does this by reducing destabilizing factors arising from health issues, such as family breakdowns, and by reducing the social upheaval caused by large-scale disease outbreaks, which improves labor availability and quality (27).

At the individual level, health insurance empowers people to take care of their own and their families' wellbeing and alleviates the economic burden imposed by diseases, or long-term treatment. Consequently, people are more able and motivated in economic activities. Specifically, health insurance cuts residents' medical service costs through dynamically adjusting policies, including deductibles, caps, funding mechanisms, reimbursement rates, and payment methods. By sharing medical expenses, health insurance alleviates the economic burden on individuals facing sudden illnesses or long-term treatments (15, 18, 28). Hence, individuals are more capable of maintaining their own and their family's health without the overwhelming pressure of medical expenses. Besides, individuals participating in healthcare insurance are more prone to receiving medical service timely, diminishing the risk of chronic diseases, thus enhancing their quality of life and facilitating engagement in social and economic activities. Moreover, in line with the life cycle theory, individuals adopt unique asset allocation strategies at various life stages, with these shifts closely connected to their health conditions and physical functions (29). With better health and less uncertainty about medical expenses, individuals can focus on career development or entrepreneurial activities. Health insurance also provides risk protection, diminishing the need for individuals to save for medical costs and boosting individual consumption capacity (30, 31).

In summary, health insurance improves health and wellbeing from both the societal and individual perspectives, promoting economic performance. On the one hand, health insurance effectively safeguards the quality of human capital, enhances productivity, and stimulates economic growth. On the other hand, healthcare insurance reduces the risks of illness, alleviates the economic burden when ill-health does arise. Furthermore, health insurance directly influences resident' consumption tendencies, reducing precautionary savings and boosting domestic demand, thereby increasing the level of consumption. Therefore, this study proposes the following hypothesis:

H1: A positive correlation exists between health insurance and economic performance.

Some studies indicate that the development of the insurance industry promotes economic growth (32–34). The promotion effect is primarily achieved through two pathways: risk transfer, reducing uncertainty and fostering consumption and research; and financial intermediation, with insurance companies investing premiums in financial markets, stimulating investment (35). Health insurance also creates progress in the health industry, boosts social welfare by increasing health expenditure, and enhances the national health level (27, 36). The beneficial role of health insurance in providing financial support for consumption is increasingly evident, leading to synchronized growth in insurance consumption and economic development (33). Health insurance, beyond typical economic impacts, collaborates with medical institutions and health management entities, providing residents with health consultation, chronic disease management, and related services. This not only improves national health and reduces illness risks and insurance payouts but also contributes to a sizable “big health industry”, fostering economic growth. Health insurance has driven the development of sectors such as pharmaceuticals, medical services, education, and older adult care, creating economies of scale and promoting economic growth. Therefore, this study proposes the following hypothesis:

H1a: Health insurance helps to promote economic growth.

Medical economic burden pertains to financial losses incurred through healthcare spending. Researchers use medical expenditure as a metric to measure the economic burden of healthcare spending. This encompasses outpatient fees, hospital charges, medication costs, and related outlays on medical services, equipment, and healthcare expenses (21). Health insurance, integral to the healthcare system, aims to alleviate residents' economic burden, ensuring the nation's right to health and life. With the broader reach of medical insurance coverage, the health insurance fund can economically compensate by sharing medical care costs, reducing the actual medical expenses for insured individuals, and alleviating their healthcare economic burden. In addition, health insurance, as a risk-sharing mechanism, spreads risk across a wider population through collected premiums. In cases of accidents or illness, it mitigates economic losses for individuals and families by offering medical services and corresponding compensation. Furthermore, health insurance negotiates reasonable medical expenses with healthcare institutions, curbing the rapid growth of healthcare costs and alleviating the economic burden. By providing medical support during illness, insurance also reduces the risk of unemployment and the loss of a wage or salary. Therefore, this study proposes the following hypothesis:

H1b: Health insurance helps to reduce the medical economic burden.

From a health economics perspective, human efforts to combat disease risks involve preventive measures and the establishment of a health insurance system. This system, as part of the health system, may support workers and boost consumer confidence. Health insurance, therefore promotes consumption through a transfer effect, leveraging the law of large numbers to reduce individual economic burdens and enhance medical service accessibility. Despite being post-event compensation, health insurance effectively increases relative income, potentially redirecting it to other consumption areas. Insurance system may also stimulate consumption through the reduced need for the precautionary savings, as it lessens the economic burden of diseases and enhances individual' ability to cope with future risks. The decreased uncertainty in future healthcare expenditures may lead people to reallocate budgets allocations, promoting current consumption. Therefore, this study proposes the following hypothesis:

H1c: Health insurance helps improve the consumption levels.

**Figure 1:**
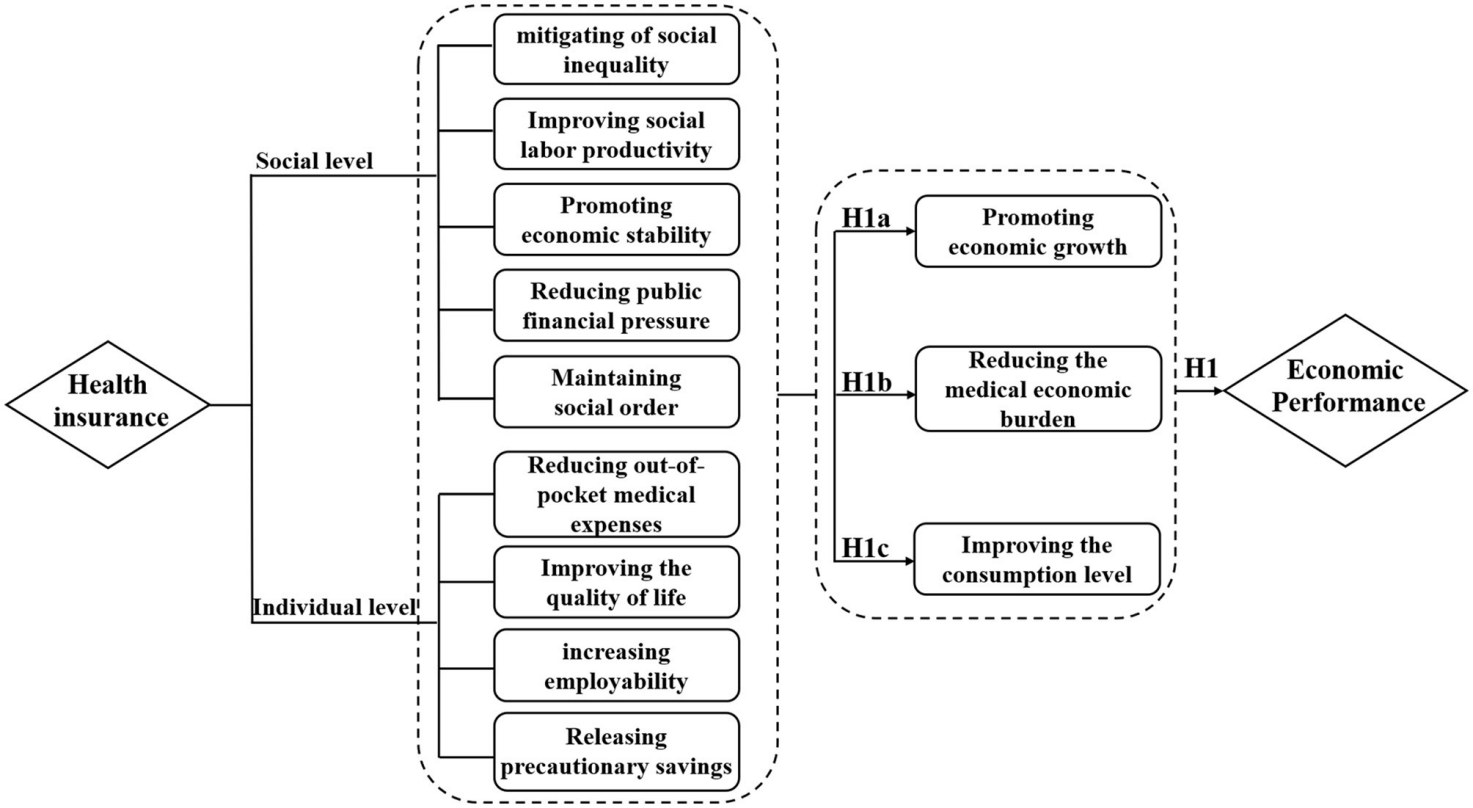
Meta-analysis diagram of the relationship between health insurance and economic performance.

Drawing on the theoretical analysis and research hypotheses above, Figure 1 illustrates the foundational framework for this study.

### 2.2 Factors that moderate the relationship between health insurance and economic performance

#### 2.2.1 Sources of difference from the sample

(1) Variations in the sampling countries. Health insurance systems in different countries are shaped by factors such as coverage scope, types of insurance, and the level of medical services. These variations contribute to diverging research outcomes regarding the relationship between health insurance and economic performance. In contrast to developing countries, developed countries typically have more robust healthcare insurance systems, abundant funds, and higher protection levels, potentially leading to a more noticeable impact on economic performance. For instance, with the United States taken as the sample source country, Raghupathi and Raghupathi concluded that there was a positive correlation between health insurance and economic performance (2). The improvement of residents' health conditions through health insurance indeed contributes to economic improvement. Beko et al. analyzed the influence of Slovenian health insurance on the national economy, revealing that it fosters national economic output and augments household income (37). Nevertheless, some studies have questioned the positive impact of health insurance on economic performance (38). Karan et al., using nationally representative data from India, found that health insurance did not significantly reduce hospitalization expenses but increased non-medical costs for households by around 5%, without alleviating the medical expenditure burden on impoverished families (39). In light of such varied research conclusions, it is imperative to discern the disparities attributable to the diverse source countries of the samples.

#### 2.2.2 Sources of difference from the measurements

(1) The variations in measuring economic performance arise from different data types. Research data, primarily including panel and cross-sectional types, can impact research results. Cross-sectional data are challenged by non-dynamism issues, while panel data, featuring multiple cross-sections over time, better address this problem and offer a more comprehensive view of variable relationships. Consequently, the utilization of different data types in existing research produces divergent research findings. For example, through the analysis of cross-sectional data, Wagstaff et al. found that health insurance did not promote economic performance (40). After analyzing panel data, Zhou et al. concluded that health insurance alleviates the financial burden on patients and promotes economic performance (41). Hence, the difference in data types may have an impact on the research results regarding the relationship between health insurance and economic performance.

(2) Different research methods are employed. Existing studies of health insurance and economic performance have primarily used difference-in-differences and various regression models such as Logit regression, Ordinary Least Squares (OLS), etc. These distinct methods come with different assumptions and applicable scopes, potentially yielding divergent results. Yang employing the difference-in-differences method, observed that the income-increasing and poverty-reduction effects of health insurance are minimal, lacking a significant impact on economic performance (10). In contrast, Wang found that healthcare insurance did enhance economic performance in a study using panel regression and quantile regression analysis (5). It is crucial, therefore to identify and understand where variations in results can be attributed to differences in the research models used.

#### 2.2.3 Sources of difference from the literature

(1) The difference in publication years. Literature published in different years is influenced by research trends, policy environments, and various factors, leading to diverse perspectives on whether health insurance promotes economic performance. In recent periods, the relationship between health insurance and economic performance has become a focal point in research, garnering attention from academia and researchers. Consequently, researchers may emphasize the positive impact of health insurance to align with academic trends. Additionally, policymakers, committed to promoting health insurance and the economy, may influence researchers to demonstrate a positive relationship, supporting policy formulation. Thus, disparities in publication years may impact the perceived link between health insurance and economic performance.

(2) Different types of literature. Various types of literature are influenced by the review process, which may result in different publication orientations for studies on the relationship between health insurance and economic performance. The literature selected for this study includes theses and journal papers. Compared to theses, journal papers undergo peer review and are more likely to publish statistically significant research. Studies yielding statistically insignificant results or challenging the prevailing literature are frequently confronted with difficulties in publication. Nevertheless, research yielding insignificant results may provide a more precise gauge of the authentic correlation between the two.

(3) The impact factors vary among different journals. Differences in journal impact factors may lead to different research orientations. Generally, compared to journals with lower impact factors, those with higher impact factors have greater academic influence and tend to prefer publishing studies with statistically significant results. In addition, with the increasing attention to the relationship between health insurance and economic performance in recent years, high-impact factor journals, to maintain the continuity of their academic influence, also emphasize the innovativeness of research conclusions. Therefore, the varying impact factors of journals may be one of the reasons for differences in the relationship between the two.

#### 2.2.4 Sources of difference from the variables

(1) The types of health insurance vary. The research samples consist of two types of health insurance: commercial health insurance and public health insurance. As an essential component of the multi-level health insurance system, commercial health insurance has rapidly developed with strong policy support. Compared to public health insurance plans, market-oriented commercial health insurance regulates the relationship between supply and demand based on voluntary participation and profit. It has significant advantages in specialized operation, management efficiency, and innovation. Commercial health insurance, characterized by “high funding and high payouts,” plays a crucial role in promoting consumer upgrading. For instance, the study of Zhao et al. indicates that commercial health insurance contributes to the construction of a multi-level healthcare system and enhances economic performance (8). Lei and Lin found that public health insurance did not reduce patients' out-of-pocket expenses (22). Given this, the different types of health insurance may lead to differences in the results of the relationship between the two.

(2) The study populations are different. Existing literature has chosen study populations from both middle-aged and older adult resident groups, as well as the entire resident population. Considering the differences in medical needs and consumption patterns, the choice of different study populations may influence the relationship between health insurance and economic performance. In the case of the middle-aged and older adult population, increasing age correlates with declining physiological functions, heightened illness risk, and subsequently increased medical expenses, placing greater economic pressure. Middle-aged and older adult individuals are more focused on preventive medical services, while the overall resident population tends to prioritize the treatment of acute illnesses. For example, Wang et al. investigated the impact of health insurance on economic performance using the middle-aged and older adult population in China as the study subjects, revealing that health insurance alleviates residents' economic burden and enhances consumption capacity (42). Liu and Zhao taking the entire resident population as the study subjects, found that health insurance did not reduce the economic burden (43).

To explore the impact of these factors on the relationship between health insurance and economic performance, this study proposes:

H2: Differences at the level of sample, measurement, literature, and variable can moderate the relationship between health insurance and economic performance.

## 3 Research method

This study employs a meta-analysis to quantitatively identify the relationship between health insurance and economic performance, along with the significance of moderating variables. Meta-analysis is a systematic and rigorous quantitative method that scientifically reviews and reanalyzes multiple quantitative results for the same research question. By collecting sample data from studies with different backgrounds, this method comprehensively evaluates research results, dissects differences among studies, and ultimately draws more accurate and robust conclusions. Therefore, it efficiently addresses issues where research findings are contentious. Additionally, this method compensates for the limitations of descriptive literature reviews in conducting quantitative analyses of research results. It demonstrates higher clarity, comprehensiveness, rigor, and systematicity in the selection, acquisition, and evaluation of original literature.

### 3.1 Study design

**Figure 2:**
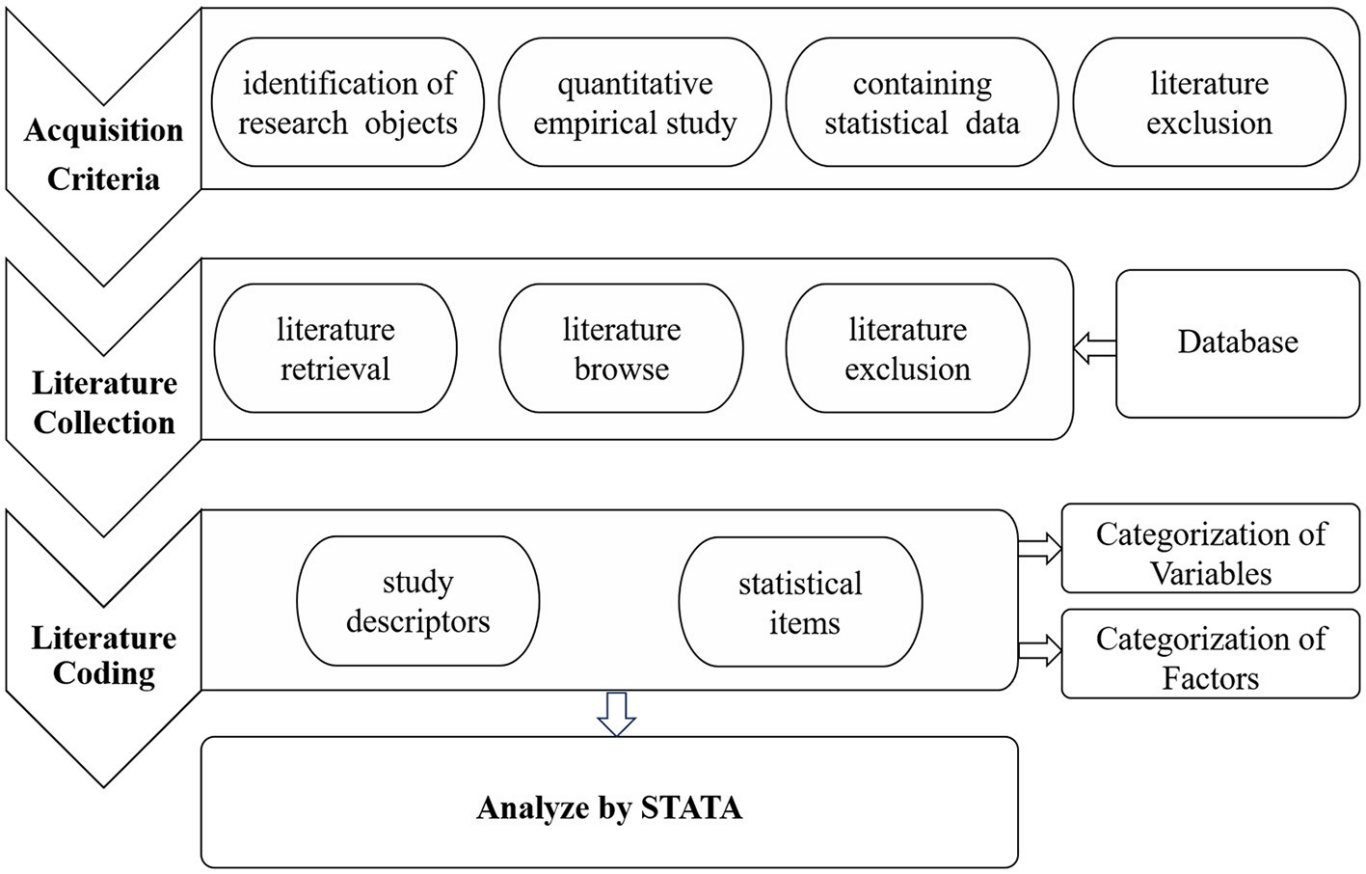
Research design of meta-analysis on the relationship between health insurance and economic performance.

Following the research objectives, research questions, and the data requirements of the meta-analysis method, this study formulated the following detailed data retrieval process. Firstly, strict literature inclusion criteria were applied, and data were gathered from various databases through searches and browsing. Secondly, relevant information and correlation coefficients were extracted from the literature and encoded, covering study description and effect value statistics. The study primarily categorizes factors influencing the health insurance and economic performance relationship. Finally, STATA was used for bias analysis, overall testing, and moderation effect testing on the data to derive research conclusions. The research design is outlined in Figure 2.

### 3.2 Criteria for selecting studies

This study performed a quantitative meta-analysis following the guidelines outlined in the Preferred Reporting Items for Systematic Reviews and Meta-Analyses (PRISMA) (44). PRISMA contributes to heightened transparency, diminishes potential biases, and avoids the redundant application of reviews, consequently enhancing both the reporting quality and methodological robustness of the meta-analysis. Functioning as a consensus-based reporting framework, PRISMA mandates that meta-analysis: (1) accumulate relevant studies, (2) categorize study characteristics, (3) compute effect sizes for each study, and (4) scrutinize features for potential moderating effects.

The criteria for select studies were based on the research question, objectives, and PRISMA requirements for methods. Specific criteria are as follows: (1) The literature has to focus on the impact of health insurance on economic performance; (2) It must be quantitative studies, excluding case analysis, theoretical and review literature; (3) The literature data need to be sufficient in terms of sample size, correlation coefficients, standard error and other convertible indicators (*t*-values, *p*-values, *z*-values, and so on); (4) Study samples should be independent, with only the latest publications retained in the case of repeated studies using the same data. Literature published in the form of theses, journal papers, and conference papers will be treated as a single study. If a literature piece involves multiple different study samples, relevant coefficients for each sample will be independently coded.

### 3.3 Literature retrieval and screening

**Figure 3:**
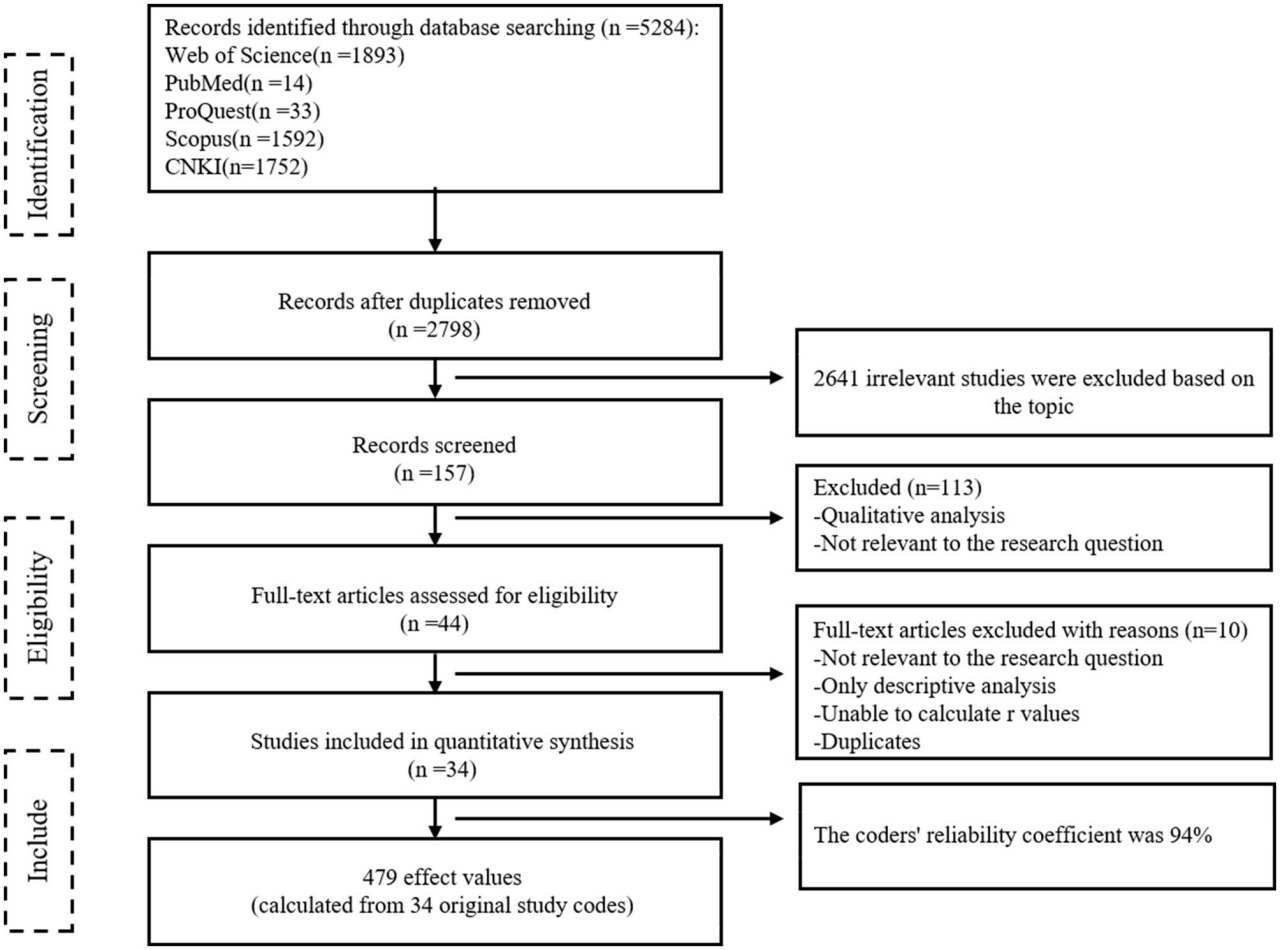
The PRISMA flow diagram of reviewing the literature.

To ensure data representativeness and completeness, this paper follows the literature selection process outlined by Havranek et al. and strictly adheres to the following steps for literature retrieval and screening (45). Firstly, the prominent databases, including PubMed, Web of Science, Scopus, CNKI, and PQDT were scrutinized. Secondly, the keywords “Health Insurance,” “Health care,” “Medicare,” “Healthcare,” “Medical care insurance,” and “Medical insurance” were combined with “Economic performance,” “Economic development,” “Economic growth,” “Economic efficiency,” “Economic benefit,” “Financial burden,” “Economic burden,” “Financial Strain,” and “empirical analysis.” Literature containing these keywords in the title or abstract was retrieved. To avoid omissions, we conducted a supplementary manual search while reviewing the literature. With a literature retrieval deadline set for October 6, 2023, a total of 5,284 literature records were collected using this process. Based on this, we further determined the literature inclusion criteria through a preliminary review of the titles, abstracts, or full texts. (1) Evaluating the research design of the literature to determine its focus on health insurance's impact on economic performance. Irrelevant studies were excluded. (2) The literature must be quantitative studies, excluding theoretical, review, case analysis, and other non-empirical studies. (3) We assess the adequacy of data by reading the full text. Literature with incomplete data was excluded. (4) Careful verification of the multiple-stage or repeated publication situations of the same sample was conducted, treating them as independent studies. Finally, 34 articles were included as analytical samples. The literature selection process for this study is shown in Figure 3.

### 3.4 Data coding rules and effect size calculation

Once the analysis sample was determined, the next step involved extracting relevant information and data from the literature. The raw data needed to be encoded and transformed to calculate key indicators for meta-analysis. To minimize coding errors, two trained coders independently encoded the 34 selected articles. The coders coded descriptive items and effect size statistics independently. Descriptive items primarily included content related to study design and literature publication, categorized into four levels: sample, measurement, literature, and variable. Sample-level descriptive items involved coding countries of origin. Measurement-level descriptive items include coding research methods and data types. Literature-level descriptive items encompassed coding information such as literature type, the impact factor, and publication year. Variable-level descriptive items coded the type of health insurance and research populations.

After converting and coding relevant information in the literature, key indicators for meta-analysis, specifically effect sizes, could be calculated. Effect size serves as a measure of the strength of the relationship between the independent variable and the dependent variable, indicating practical significance. In this study, the effect size generally corresponded to the correlation coefficient between variables, as the included literature used correlation coefficients or regression techniques for analysis. Therefore, an effect size based on the correlation coefficient (*r*-based) was used to represent the relationship between health insurance and economic performance. According to the formula provided by Rosenthal (46), the estimation parameters (*t*-values) of the original studies are converted into correlation coefficients (*r*) using the formula: *r* $=\sqrt{\;[t^{2}/(t^{2}+\;df)]}$). Here, df is the degree of freedom related to the *t*-value, which can be calculated based on the sample size and variable values in the original study.

To account for variations in sample size among the original studies, the correlation coefficient (*r*) was further transformed into the standard effect size of Fisher's *Z*. The standard error of *Z* (SEz) was then calculated to correct for the bias resulting from differences in sample size. The specific calculation method for standard error is as follows: (1) Z_r_ = 0.5ln [(1+r)/(1–r)]; (2) Calculate the variance of Z, Vz = 1/(n−3); (3) Calculate ${\text{SE}}_{\text{z}}=\sqrt{V_{Z}}$.

The above calculation process was performed using Stata17 software. The unit of analysis for effect sizes was independent samples. If multiple independent samples exist in the original study, they were coded multiple times. The final consistency coefficient for coding was 94% (>90%), affirming the high reliability of this coding method and supporting the credibility of the results (47). Ultimately, we extracted 479 effect sizes from the 34 empirical studies.

## 4 Results of meta-analysis

### 4.1 Publication bias analysis

It is necessary to assess publication bias to ensure the accuracy and reliability of the results before conducting the meta-analysis. The current methods for testing publication bias mainly include the funnel plot method, fail-safe number method, Egger's test, Begg's method, and Trim method. This study employed the first two methods to assess publication bias.

**Figure 4:**
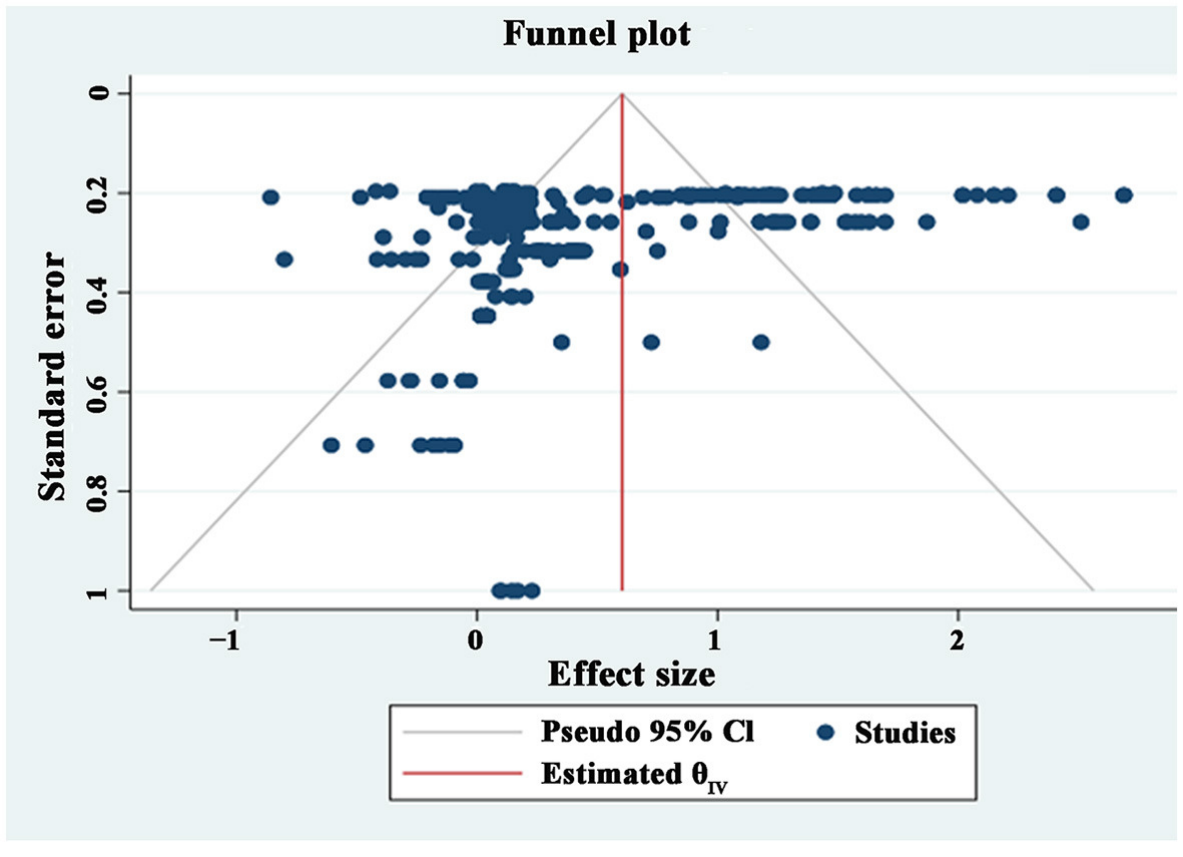
Funnel plot.

As shown in Figure 4, the funnel plot of the sample literature included in the meta-analysis is presented. The distribution of effect sizes is mainly concentrated at the top of the funnel plot, spreading on both sides of the overall effect size, forming an inverted funnel shape. However, there is a small number of effect sizes that are not evenly distributed. Therefore, relying solely on visual inspection to determine the existence of publication bias is not precise enough and additional methods are needed for a comprehensive judgment.

The fail-safe number method is a quantitative method for testing publication bias. As shown in Table 1, the fail-safe value in this study is 321,170, significantly higher than the “5 k + 10” criterion (2,405). Furthermore, the fail-safe values for other sub-dimensions also passed the test, indicating unbiased and robust results.

**Table 1:** Test of publication bias.

**Category**	**Sample size**	**Fail-safe number**	
	***K***	**Threshold**	***N*_fs0.05_**
All	479	2,405	321,170
Economic growth	128	650	126,497
Reducing medical economic burden	213	1,075	13,954
Improving the consumption level	138	700	8,611

### 4.2 Overall test

This study conducts an overall test on the effect sizes and the standard errors, including a heterogeneity test and the model results evaluation. Effect sizes were combined to comprehensively assess the reliability of the hypothesis about the relationship between health insurance and economic performance. Q-statistics, significance level, and *I*^2^ are used to evaluate the heterogeneity (48). When *Q* > df (*Q*), *p* < 0.05, and *I*^2^ > 50%, the results of various studies are considered heterogeneous. In these cases, a random-effects model should be chosen to combine effect values; for others, a fixed-effects model is selected.

Table 2 shows the results of the overall test of health insurance and economic performance. According to the heterogeneity test results, *Q* = 3,229.21 > 478, *P* < 0.05, and *I*^2^ = 85.20%, indicating high heterogeneity among the 478 effect sizes in the meta-analysis. True differences in the effect sizes and random errors accounted for 85.20 and 14.80% of the observed variation, respectively. Therefore, a random-effects model needed to be selected. The variance value is 0.322, suggesting that 32% of the variation can be utilized to calculate weights.

**Table 2:** Meta-analysis results of the health insurance and economic performance.

**Variable**	**Heterogeneity test**				**Effects model**					**The magnitude of a correlation**
	**Df**	***P***	***I*^2^(%)**	***Q***	***z***	**Variance**	**Point estimation**	**Lower limit**	**Upper limit**	
All	478	0.000	85.20	3,229.21	15.74	0.322	0.429	0.381	0.475	Large
Economic growth	127	0.000	88.43	1,097.86	17.17	0.353	0.746	0.693	0.791	Large
Reducing medical economic burden	212	0.000	74.97	846.94	6.73	0.205	0.242	0.173	0.308	Medium
Improving the consumption level	137	0.000	39.21	225.37	15.73	1.650	0.295	0.260	0.329	Medium

The model testing results showed the correlation coefficient between health insurance and economic performance was 0.429, with a 95% confidence interval of (0.381, 0.475). Gignac and Szodorai proposed guidelines to interpret the magnitude of a correlation (49). In magnitude, values of 1 < *r* < 0.2, 0.2 < *r* < 0.3, and *r* > 0.3 are considered small, medium, and large, respectively. The correlation coefficient between health insurance and economic performance in this study exceeded 0.3, confirming a robust positive correlation and supporting Hypothesis 1. The correlation coefficient for economic growth, reducing medical economic burden, and improving the consumption level were 0.485, 0.159, and 0.258, respectively. All coefficients are significant at the 95% confidence interval (*p* < 0.05). This suggests that health insurance has a positive impact on economic growth, reducing medical economic burden, and improving the consumption level. Hypotheses H1a, H1b, and H1c are all validated. The relationship between health insurance and economic performance was highly positive, while the relationship between health insurance with reducing medical economic burden and improving the consumption level was moderately positive.

Meta-analysis allows for a scientific and precise assessment of the relationship between health insurance and economic performance. In addition, the first research question in this study can be answered: Does health insurance promote economic performance? However, it is noteworthy that the distribution of effect size for the relationship between the two varies significantly among research reports. There may be some moderating factors influencing the intensity of effect size, necessitating further tests to identify potential inconsistencies reason.

### 4.3 Moderation effect test

The overall effect test of the meta-analysis indicates high heterogeneity between health insurance and economic performance, suggesting that the relationship between the two is influenced by potential moderating variables. To test this effect, we coded and conducted subgroup analyses on the collected data. Table 3 presents the subgroup analysis results. Specifically: (1) There was a significant difference in the relationship between health insurance and economic performance when using samples from developed countries or developing countries (*p* < 0.05). Moreover, the promotion effect of health insurance on economic performance is stronger in developed countries (*r* = 0.669) compared to developing countries (*r* = 0.297). (2) There was a significant difference in the research results on the relationship between the two when using panel data and cross-sectional data (*p* < 0.05). Subgroup analysis shows that the relationship between health insurance and economic performance was significantly higher in panel data (*r* = 0.498) than in cross-sectional data (*r* = 0.188). Furthermore, the research method had a significant moderating effect on the relationship between the two (*p* < 0.05). Compared to non-regression methods (*r* = 0.334), regression methods (*r* = 0.462) showed a stronger positive correlation. (3) From the literature perspective, publication year, literature type, and the impact factor can all moderate the relationship between health insurance and economic performance (*p* < 0.05). Subgroup analysis indicated that health insurance's promotion effect on economic performance was stronger for publications after 2010 (*r* = 0.431) than before 2010 (*r* = 0.358). Compared to theses (*r* = 0.202), journal papers (*r* = 0.502) showed a stronger positive correlation. Additionally, a higher impact factor of the journal correlated with a more positive relationship between health insurance and economic performance. (4) From the variable perspective, the type of health insurance and the research populations can significantly moderated the relationship between the two (*p* < 0.05). Public health insurance (*r* = 0.466) has a stronger promotion effect on economic performance than commercial health insurance (*r* = 0.297). The relationship between health insurance and economic performance was significantly higher in studies with the overall research population (*r* = 0.489) than in studies with the middle-aged and older adult as the research population (*r* = 0.092).

**Table 3:** Meta-analysis results of the moderating effects.

	**Variables**	**Category**	***K***	**95%CI**			**Heterogeneity test**		
				**Estimated value**	**Lower limit**	**Upper limit**	***Q***	**Df**	***P***
Sample	Country	Developed country	133	0.669	0.595	0.731	1,575.38	132	0.000
		Developing country	339	0.297	0.250	0.343	1,081.90	338	
Measurement	Data type	Panel	352	0.498	0.445	0.547	2,643.90	351	0.000
		Cross-sectional	127	0.188	0.107	0.266	378.57	126	
	Research method	Regression method	348	0.462	0.407	0.514	2,596.01	347	0.000
		Other methods	117	0.334	0.233	0.427	576.87	116	
Literature	Publication year	≤ 2010	13	0.358	0.037	0.613	86.25	12	0.000
		>2010	466	0.431	0.383	0.477	3,142.70	465	
	Literature type	Thesis	144	0.202	0.141	0.261	157.25	143	0.000
		Journal	335	0.502	0.449	0.551	2,955.13	334	
	Impact factor	≤ 5	108	0.391	0.296	0.478	580.48	107	0.000
		>5	147	0.659	0.588	0.720	1,770.02	146	
Variable	Health insurance type	Commercial health insurance	99	0.297	0.240	0.353	182.76	98	0.000
		Public health insurance	380	0.466	0.410	0.519	2,946.82	379	
	Research populations	Middle-aged and older adult	100	0.092	0.013	0.170	186.43	99	0.000
		Overall	368	0.489	0.438	0.537	2,685.48	368	

In summary, the country of sample origin, data type, research method, publication year, literature type, journal impact factor, type of health insurance, and research populations all presented significant moderating effects regarding the relationship between health insurance and economic performance (*p* < 0.05). Therefore, hypothesis H2 was supported.

### 4.4 Robustness test

Drawing on the research of Tilley et al. (50), this paper applied the meta-regression analysis method to test the robustness of the moderation effect results mentioned above. As indicated in Table 4, the regression coefficients at the sample level, measurement level, literature level, and variable level were all positive, and the results are highly significant (*p* < 0.05). Hypothesis H2 was validated. These findings align with the subgroup analysis, confirming the robustness of the meta-analysis results.

**Table 4:** Robustness test.

	**Moderating variables**	***N***	***B***	**SE**	***T***	***P***	**σ**	***I* ^2^**
Sample	Country	133/339	0.810	0.048	17.06	0.000	0.310	0.807
Measurement	Data type	352/127	0.550	0.032	17.26	0.000	0.286	0.828
	Research method	348/117	0.501	0.034	14.77	0.000	0.315	0.840
Literature	Publication year	13/466	0.376	0.169	2.23	0.026	0.310	0.839
	Literature type	144/335	0.152	0.058	2.60	0.010	0.290	0.833
	Impact factor	108/147	0.413	0.066	6.31	0.000	0.400	0.892
Variable	Health insurance type	99/380	0.271	0.062	4.39	0.000	0.301	0.833
	Research populations	100/368	0.453	0.073	6.25	0.000	0.281	0.822

## 5 Discussion

### 5.1 Relationship between health insurance and economic performance

There have been diverse perspectives and research outcomes around the relationship between health insurance and economic performance, but no research has clarified these variations. This study applied meta-analysis to evaluate the overall relationship between the two, revealing a strongly positive correlation. It indicates that health insurance positively influences economic performance, supporting the first perspective and clarifying the debate about the direction of relationship and magnitude. This study contradicts the view of a non-existent or negative correlation between health insurance and economic performance, underlining the statistically significant relationship that shouldn't be ignored or overstated in practice.

Specifically, health insurance positively influences economic growth, alleviates economic burdens, and enhances consumption capabilities in the process of promoting economic performance. A crucial aspect of the relationship between health insurance and economic performance is the factor of workers' productivity (51). Poor individual health may lead to the loss of labor and productivity. The health insurance system, by improving residents' health, enhances labor capabilities, efficiency, and quality of life, effectively ensuring the quality of human capital and subsequently improving economic performance. With the development of health insurance, more funds are allocated to research and development of new medical technologies, equipment, and treatment methods. These innovations increase productivity in the healthcare industry, reduce medical costs, enhance the quality of medical services, and consequently drive the development of the healthcare sector, contributing to economic growth. Additionally, health insurance, through its risk transfer function, reduces uncertainty, encourages individual consumption, and promotes increased business investments, leading to economic growth. Moreover, through its economic compensation function, health insurance reduces out-of-pocket medical expenses for insured individuals, thereby alleviating their economic burden. Through widespread adoption and promotion of health insurance and the guidance it provides to individuals, people can make more rational use of healthcare resources, avoiding waste and excessive consumption. This not only helps in lowering healthcare costs but also enhances the efficiency of healthcare resource utilization, further mitigating the economic burden. Furthermore, health insurance stabilizes future expectations by alleviating concerns about medical expenses, reducing the need for precautionary savings. This allows redirected funds for higher-level consumption, enhancing people's quality of life and boosting related industries, contributing to economic growth. With reduced their expenditure on their healthcare, people have more disposable income for other consumption. This increased disposable income enhances people's consumption capabilities, driving the growth of goods and services sales, and further stimulating economic growth.

### 5.2 Moderation effect analysis

It is essential to note that the overall conclusions drawn from the meta-analysis only focus on the correlation between two variables and do not invalidate studies that lack support. The correlation degree of the relationship between health insurance and economic performance may also be affected by other variables. This study conducted subgroup analyses at the sample, measurement, literature, and variable levels, systematically investigating potential moderating factors influencing the relationship between the two. Specific investigation results revealed several points:

At the sample level, studies conducted in developed countries show a stronger positive effect of health insurance on economic performance compared to studies in developing countries. This was mainly related to human capital, as healthier individuals are more effective in both physical and mental labor. In developed countries where labor is scarce, the impact of health insurance on human capital efficiency is more apparent. Furthermore, developed countries generally have more robust health insurance systems with comprehensive management and regulatory mechanisms, ensuring service quality and efficiency. Residents can access higher-quality medical services, leading to higher labor efficiency. In contrast, developing countries may lack robust management and regulatory systems, resulting in lower healthcare standards. These factors make the positive impact of health insurance on economic performance more pronounced in developed countries.

At the measurement level, this study found that using panel data yielded a stronger positive effect of health insurance on economic performance than using cross-sectional data. Panel data analysis provides a dynamic analysis of the development of health insurance, offering more comprehensive data and more robust results. Cross-sectional analysis, which collects data at a specific point in time, may yield less accurate conclusions, even with representative samples, potentially leading to bias. Additionally, employing regression methods results in a stronger positive effect of health insurance on economic performance compared to other methods. This suggests that the research method influences the study's outcomes. Regression analysis can more accurately describe the complex relationship between the two by setting variables and establishing mathematical models. Moreover, the results of regression analysis can be verified through statistical methods to ensure reliability and accuracy. Non-regression analysis results may lack such verification mechanisms, reducing their relative reliability. Therefore, regression analysis can more accurately describe and handle the relationship between health insurance and economic performance. Thus, this study concluded that regression analysis was more effective than non-regression analysis in capturing the promoting effect of health insurance on economic performance.

At the literature level, the publication year, type of literature, and journal impact factors significantly moderated the relationship between health insurance and economic performance. In terms of the publication year, more recent publications exhibited a stronger positive effect of health insurance on economic performance. This implies continuous improvements in health insurance, providing essential support for economic development. Literature published in high-impact journals demonstrated a more pronounced positive effect of health insurance on economic performance than literature in low-impact journals. This highlights the emphasis of high-impact journals on paper quality and the statistical significance of results. Studies published in journals, compared to theses, exhibit a stronger positive effect of health insurance on economic performance. This distinction may stem from the peer review process, which tends to view a positive correlation between the two as having favorable policy implications.

At the variable level, this study identifies significant moderating effects of health insurance type and research populations on the relationship between health insurance and economic performance. Health insurance types include commercial health insurance and public health insurance. Public health insurance exhibits a more pronounced positive effect on economic performance compared to commercial health insurance. This could be attributed to public health insurance having a wider reach, substantially reducing the overall medical economic burden and accruing more human capital, thereby fostering economic growth. Regarding research populations, existing literature mainly focused on middle-aged and older adult populations and the overall populations. Literature concentrating on the middle-aged and older adult population might not fully capture the entire developmental trajectory of health insurance, thereby weakening its impact on economic performance. Conversely, literature focusing on the overall population maximizes the comprehensive advantages of health insurance, showcasing the maximum impact on economic performance. Hence, in comparison to literature focusing on the middle-aged and older adult populations, literature concentrating on the overall populations concludes that health insurance has a more robust positive effect on economic performance. The research results are summarized in Table 5.

**Table 5:** Meta results of various variables.

**Variables**	**Category**	**Subdimension**	**Hypothesis**	**Significance**	**The magnitude of a correlation**
				**Yes/no**	
Core variables and the subdimensions	Overall		H1	Yes	Large
	/	Economic growth	H1a	Yes	Large
	/	Reducing medical economic burden	H1b	Yes	Medium
	/	Improving the consumption level	H1c	Yes	Medium
Moderating variables and the subdimensions	Country	Developed country	H2	Yes	Large
		Developing country			Medium
	Data type	Panel		Yes	Large
		Cross-sectional			Small
	Research method	Regression method		Yes	Large
		Other methods			Large
	Publication year	≤ 2010		Yes	Large
		>2010			Large
	Literature type	Thesis		Yes	Medium
		Journal			Large
	Impact factor	≤ 5		Yes	Large
		>5			Large
	Health insurance type	Commercial health insurance		Yes	Medium
		Public health insurance			Large
	Research populations	Middle-aged and older adult		Yes	Small
		Overall			Large

## 6 Conclusions

As disease patterns evolve and population aging intensifies, it is crucial to consider the issue of poverty caused by illness. Health insurance serves as a vital instrument for addressing the problem of illness-induced poverty, enhancing residents' health, bolstering the labor force, and boosting productivity. In the ongoing evolution and enhancement of the health insurance system, the pivotal question of whether health insurance safeguards residents' health while concurrently promoting economic performance has emerged as a critical focus for optimizing institutional efficacy. Moreover, evaluating the economic performance of health insurance is essential for enhancing the coordination of the medical security system and achieving sustainable development of the economy and society.

The results of this study indicated a significant positive correlation between health insurance and economic performance, supporting H1. Furthermore, this study clarifies that the correlation between the two is strongly positive. This suggests that health insurance has significant economic spillover effects. Policymakers should deepen the reform of the healthcare system, to strengthen poverty reduction and income increasing effects of health insurance. They should aim to establish a more equitable, mature, and sustainable healthcare system.

Additionally, this study employs moderation effect tests to delve deeper into the relationship between health insurance and economic performance. The source country of the sample, data type, research method, publication year, type of literature, impact factor, health insurance type, and research populations exhibit moderating effects on this relationship. It is found that public health insurance has a greater impact on economic performance than commercial health insurance. Consequently, it is advisable to judiciously balance the proportions of public and commercial health insurance, progressively elevating the overall standard to establish a high-quality universal healthcare system. Moreover, policymakers should intensify support for pivotal groups and sectors, refining the precision of health insurance. For example, efforts should be made to expand the coverage of health insurance for the older adult, ensure medical security for vulnerable groups, and actively respond to the challenges of an aging population.

This study explores the relationship between health insurance and economic performance, yielding comprehensive and objective conclusions. By clarifying this relationship, the foundation is laid for establishing an optimal health insurance framework that, enhances economic wellbeing. Moreover, diversifies health insurance studies but also provides ample empirical evidence for ongoing improvements in social human capital efficiency, fostering sustainable economic development. Simultaneously, the precise identification of the magnitude of the correlation between the two serves as a scientific cornerstone for informed decision-making, guiding the formulation of judicious and effective health insurance policies.

## 7 Limitations

This study systematically reviews existing literature through meta-analysis, examining the relationship between health insurance and economic performance. It validates and gauges the strength of this relationship, overcoming biases in prior individual studies to draw relatively comprehensive conclusions. However, this study still has some limitations. Firstly, it only considers empirical testing literature, neglecting qualitative sources like case studies in the meta-analysis. Secondly, the multifaceted nature of the relationship between health insurance and economic performance remains incompletely explored, with certain influencing factors unaddressed. For example, the inclusion of different types of literature can impact the relationship between health insurance and economic performance. In the future, efforts can be made to overcome methodological limitations by including more non-empirical literature for analysis, thereby obtaining more accurate research results. Moreover, the results of the meta-analysis depend on the quality of the encompassed studies. Should these studies exhibit methodological shortcomings, biases, or inadequate statistical power, the findings of the meta-analysis could be compromised. Future research can address our shortcomings in the following ways: (1) Exploring new methods to study the relationship between health insurance and economic performance, such as adopting systematic literature reviews and qualitative meta-analyses. Overcoming methodological limitations by including more non-quantitative literature for analysis can lead to more accurate research results. Using network analysis and CiteSpace-based bibliometric analysis to visualize and interpret the structure and dynamics of research on health insurance and economic performance. Additionally, citation analysis, a bibliometric method using extensive citation data, reveals connections and patterns in the literature, aiding a deeper understanding of current research trends. (2) The multifaceted nature of the relationship between health insurance and economic performance remains incompletely explored, with certain influencing factors unaddressed. Future research could delve into the impact of intermediary variables, such as innovation elements, on this relationship. (3) Given the critical connection between health insurance and economic performance, it is profitable to investigate their route in fostering sustainable economic development.

## Data availability statement

The raw data supporting the conclusions of this article will be made available by the authors, without undue reservation.

## Author contributions

CF: Conceptualization, Data curation, Formal analysis, Writing – original draft, Writing – review & editing. CL: Data curation, Methodology, Writing – review & editing. XS: Funding acquisition, Writing – review & editing.
